# Maternal obesity and gestational diabetes reprogram the methylome of offspring beyond birth by inducing epigenetic signatures in metabolic and developmental pathways

**DOI:** 10.1186/s12933-023-01774-y

**Published:** 2023-03-04

**Authors:** Juan José Alba-Linares, Raúl F. Pérez, Juan Ramón Tejedor, David Bastante-Rodríguez, Francisco Ponce, Nuria García Carbonell, Rafael Gómez Zafra, Agustín F. Fernández, Mario F. Fraga, Empar Lurbe

**Affiliations:** 1 Cancer Epigenetics and Nanomedicine Laboratory, Nanomaterials and Nanotechnology Research Center (CINN-CSIC), University of Oviedo, Oviedo, Spain; 2 Health Research Institute of Asturias (ISPA-FINBA), University of Oviedo, Oviedo, Spain; 3 Institute of Oncology of Asturias (IUOPA), University of Oviedo, Oviedo, Spain; 4 Department of Organisms and Systems Biology (B.O.S.), University of Oviedo, Oviedo, Spain; 5 Biomedical Research Networking Center on Rare Diseases (CIBERER), Institute of Health Carlos III (ISCIII), Madrid, Spain; 6 Health Research Institute INCLIVA, Valencia, Spain; 7 Biomedical Research Networking Center for Physiopathology of Obesity and Nutrition (CIBEROBN), Institute of Health Carlos III (ISCIII), Madrid, Spain; 8 Servicio de Pediatría, Consorcio Hospital General Universitario de Valencia, Valencia, Spain

**Keywords:** Metabolism, DNA methylation, Epigenetics, Obesity, Gestational diabetes, Newborn, Development, Longitudinal

## Abstract

**Background:**

Obesity is a negative chronic metabolic health condition that represents an additional risk for the development of multiple pathologies. Epidemiological studies have shown how maternal obesity or gestational diabetes mellitus during pregnancy constitute serious risk factors in relation to the appearance of cardiometabolic diseases in the offspring. Furthermore, epigenetic remodelling may help explain the molecular mechanisms that underlie these epidemiological findings. Thus, in this study we explored the DNA methylation landscape of children born to mothers with obesity and gestational diabetes during their first year of life.

**Methods:**

We used Illumina Infinium MethylationEPIC BeadChip arrays to profile more than 770,000 genome-wide CpG sites in blood samples from a paediatric longitudinal cohort consisting of 26 children born to mothers who suffered from obesity or obesity with gestational diabetes mellitus during pregnancy and 13 healthy controls (measurements taken at 0, 6 and 12 month; total N = 90). We carried out cross-sectional and longitudinal analyses to derive DNA methylation alterations associated with developmental and pathology-related epigenomics.

**Results:**

We identified abundant DNA methylation changes during child development from birth to 6 months and, to a lesser extent, up to 12 months of age. Using cross-sectional analyses, we discovered DNA methylation biomarkers maintained across the first year of life that could discriminate children born to mothers who suffered from obesity or obesity with gestational diabetes. Importantly, enrichment analyses suggested that these alterations constitute epigenetic signatures that affect genes and pathways involved in the metabolism of fatty acids, postnatal developmental processes and mitochondrial bioenergetics, such as *CPT1B*, *SLC38A4*, *SLC35F3* and *FN3K*. Finally, we observed evidence of an interaction between developmental DNA methylation changes and maternal metabolic condition alterations.

**Conclusions:**

Our observations highlight the first six months of development as being the most crucial for epigenetic remodelling. Furthermore, our results support the existence of systemic intrauterine foetal programming linked to obesity and gestational diabetes that affects the childhood methylome beyond birth, which involves alterations related to metabolic pathways, and which may interact with ordinary postnatal development programmes.

**Supplementary Information:**

The online version contains supplementary material available at 10.1186/s12933-023-01774-y.

## Background

Obesity is a negative health condition characterized by an excessive accumulation of fat that is diagnosed at a body mass index (BMI) equal to or greater than 30 kg/m^2^ [1]. In recent decades, obesity has become a central public health concern due to its increasing prevalence, which has reached worldwide pandemic levels, and because it represents a leading risk factor for the development of cardiovascular, metabolic diseases (e.g., hypertension, myocardial infarction, stroke, diabetes mellitus) and certain types of cancer, amongst other illnesses [1, 2].

Interestingly, epidemiological studies have shown how maternal obesity during pregnancy constitutes an important risk factor related to the appearance of cardiometabolic diseases in the offspring [3], including obesity, elevated blood pressure, impaired insulin/glucose homoeostasis, increased inflammatory markers and altered lipid profiles [4–8]. This is a serious concern since prevalence rates of obesity in pregnant women can exceed 30% [9]. Furthermore, a recent longitudinal girl study with a follow-up from birth to 10 years has found that birth weight and maternal obesity are the main risk factors responsible for the appearance of obesity at 5 years, while at 10 years the only significant related condition is maternal obesity [10].

The increased risk for these conditions is maintained not only in childhood, but also in terms of adulthood morbidity and mortality [11–13], which evinces profound implications for the design of public health policies and interventions, especially in the case of cardiovascular diseases. Moreover, the most common medical complication of pregnancy, gestational diabetes mellitus (GDM, hyperglycaemia that develops during pregnancy and resolves after birth), represents another risk factor for the development of obesity and cardiovascular disease in both the mother and child [14]. A recent cohort study has evinced that previous GDM leads to a higher incidence of dyslipidemia in women [15]. What is more, maternal obesity is also correlated with metabolic complications in pregnancy such as GDM, gestational hypertension and pre-eclampsia, highlighting the fact that obesity and GDM are very intertwined [16].

However, due to obesity's multifactorial nature [17], little is known about the molecular mechanisms that underlie these strong epidemiological findings. Furthermore, it remains unclear whether childhood obesity is simply the result of the unhealthy eating behaviour instilled by parents during postnatal growth or whether the intrauterine environment may be capable of affecting children of obese mothers, predisposing them to the development of cardiometabolic diseases. In line with the theory of the developmental origins of health and disease, the intrauterine period is crucial to understand the adult risk to experience cardiovascular events [18], but at the same time, early postnatal development has gained relevance in recent decades. Longitudinal studies which trace a life course perspective are needed to address these questions, exploring the interrelations between the influence of the intrauterine environment and developmental processes [19].

To date, the mechanisms governing the influence of the intrauterine environment on the offspring’s health are still under discussion. For instance, extracellular vesicles are posited to play roles in the systemic regulation of physiological processes and pathologies [20]. In this line, we propose that epigenetic remodelling, which is especially sensitive to extrinsic and intrinsic influences during early life [21, 22], could constitute the molecular mechanism through which intrauterine stimuli can affect the biology of the cell. In fact, it is well known that DNA methylation patterns are key regulators of genes involved in pancreatic β-cell homeostasis, including insulin signalling and secretion [23]. At the same time, metabolic imbalance can disrupt epigenetic mechanisms through alterations in the levels of tricarboxylic acid cycle intermediates and the redox balance, which subsequently can have an impact on gene regulation and DNA damage and repair [24]. Ten-eleven translocation methylcytosine dioxygenases, responsible for DNA demethylation processes, are sensitive to these metabolic dysfunctions, such as obesity and diabetes mellitus, establishing a crosstalk between metabolism, epigenetics and genome stability [24]. In agreement with this, gestational diabetes [25, 26], maternal obesity [27] and hypertension [19] have been consistently associated with DNA methylation alterations in placenta, blood and tissue samples from offspring. To shed light on this issue, longitudinal studies are of great value for several reasons, among them: (a) in the field of developmental biology, they allow the definition of the key time frame when epigenetic alterations are most dynamic, and therefore, when external stimuli can have the greatest influence at the molecular level; (b) they can define epigenetic patterns that are acquired through uterine exposure but are maintained over time, acting as reliable childhood biomarkers.

This study presents, to the best of our knowledge, the first longitudinal genome-wide analysis of the methylome of whole blood samples from a paediatric cohort of children born to mothers suffering from obesity or obesity with GDM during pregnancy and healthy controls. We performed longitudinal measurements throughout the first year of life (0, 6 and 12 months) on 39 subjects (total N = 90) using Illumina Infinium MethylationEPIC BeadChip arrays to profile more than 770,000 CpG sites. The design allowed us to carry out both cross-sectional and longitudinal analyses in order to derive DNA methylation alterations associated with developmental and pathology-related epigenomics.

## Methods

### Selection of study subjects

This cohort is part of a prospective and ongoing study begun in 2018. The present research includes data from April 2018 to February 2020. Newborns born at term (gestational age ≥ 37 weeks) at the General Hospital of Valencia were randomly recruited to participate in the study. At or before birth, all parents gave informed consent for their children to participate in the study, which was approved by the Clinical Research Ethics Committee of the Consorcio Hospital General Universitario de Valencia.

Three groups were established on the basis of mothers’ BMI and the presence or absence of GDM: children of obese mothers with GDM, children of obese mothers without diabetes and children of control mothers (normal weight BMI 18.5–24.9 kg/m^2^, without pathology). Obesity in pregnant women was defined as BMI ≥ 30 kg/m^2^ at the beginning of the pregnancy. The screening of GDM consisted of a 50 g oral glucose load (glucose challenge test or GCT) followed by a plasma sugar level test 1 h later when the women’s pregnancies were at between 24 and 28 weeks of gestation. A level of more than 7.8 mmol/L (140 mg/dL) indicated the need for full diagnostic testing with an oral glucose tolerance test (OGTT, 100 g oral glucose load, testing during 3 h). The diagnosis of GDM required any two of the four plasma glucose values to be equal to or above the following values: (a) after overnight fast: 105 mg/dL (5.8 mmol/L); (b) 1 h: 190 mg/dL (10.6 mmol/L); (c) 2 h: 165 mg/dL (9.6 mmol/L); (d) 3 h: 145 mg/dL (8.1 mmol/L).

The exclusion criteria employed were: multiple gestations, overweight (rather than obese) women (BMI 25.0–29.9 kg/m^2^), underweight women (BMI < 18.5 kg/m^2^) and any complication during gestation apart from GDM. The general characteristics of gestation and delivery were obtained from routine obstetric records. The subjects were followed-up at the General Hospital of Valencia Outpatient Clinic for the first year of the child’s life.

### Anthropometric parameters

At birth and during the follow-up, weight and length were measured by trained nurses. Length was measured in the supine position using a paediatric measuring device. Weight was measured in the Maternity Unit using an ADE scale model M112600 (GmbH & Co.) and in the Outpatient Clinic using a Seca 354 scale (GmbH & Co.). Body mass index (BMI) was calculated as the weight in kilograms divided by the square of the height in meters for mothers and using WHO AnthroPlus software for children.

### Sample collection, DNA extraction and genome-wide DNA methylation analysis

Blood samples were taken from 39 subjects at three time points (birth, 6 and 12 months of age; total N = 90). At birth, samples were collected from the umbilical cord, while at 6 and 12 months they were taken from peripheral venous blood.

Genomic DNA was extracted from whole blood cells with the RealPure kit (RealPure, REAL, Durviz) and quantified with the Nanodrop-2000C Spectrophotometer. Next, the DNA was bisulphite converted using the EZ-96 DNA Methylation Kit conversion protocol (Zymo Research). Finally, the Illumina Infinium HD Methylation Assay protocol was performed by hybridising processed DNA samples to Infinium MethylationEPIC BeadChips.

### Array data preprocessing

All MethylationEPIC BeadChip data analyses were performed using the statistical software R (v.4.0.2). First, IDAT files were imported and processed with the *minfi* package (v.1.32.0) [28]. Self-reported sex and subject genetic tracking were validated by accessing the array methylation data for sex chromosome probes and SNP probes, using the *getSex* and *getSnpBeta* functions from *minfi*. In addition, probes from *sesame* package (v.1.4.0) [29] were used to carry out ethnic inference analysis and ensure correct sample tracking. All samples passed the specific quality control of the *minfi* package for intensity signals both in methylated and unmethylated channels.

Probes were filtered out if: (a) detection p-value was > 0.01 in any sample; (b) they were located in sex chromosomes; (c) they were cross-reactive or multi-mapping [30, 31] and (d) they included SNPs with MAF ≥ 0.01 at their CpG or SBE sites (dbSNP v.147). Moreover, clustered-distribution analysis using the *gaphunter* function (threshold = 0.25, outCutoff = 5/90) of the *minfi* package allowed the detection of experiment-specific conflicting probes (N = 1,065), which were discarded for downstream analysis [32]. After this, intensity values were subjected to background correction using the ssNoob method [33] in *minfi* and extracted β-values were normalised using the BMIQ approach [34] implemented in *ChAMP* (v.2.16.2) [35]. The final number of probes that passed through all the filters was 772,088.

### Cell-type deconvolution

Cell-type composition was predicted from DNA methylation data by the Houseman algorithm [36] implemented in the *ENmix* package (v.1.28.2) [37]. Appropriate and specific reference datasets were used for the cell-type prediction of cord-blood *FlowSorted.CordBloodCombined.450 k* [38] and peripheral-blood samples *FlowSorted.Blood.EPIC* [39].

### Probe-level differential methylation analyses

First, β-values were logit-transformed to M-values with the *beta2m* function of the *lumi* package (v.2.40.0) in order to achieve greater homoscedasticity in the differential methylation analyses [40]. Then, linear mixed models were built using the *limma* package (v.3.44.3) [41] to detect differentially methylated probes (DMPs). Several statistical models were designed by fitting M-values as the dependent variable. All models included fixed covariates that accounted for possible experimental batch effects (array plate), sex and cell-type composition from deconvolution analyses, while subject-specific contributions were controlled via random-effects components. Cross-sectional comparisons were performed using *Group* (Control, Obesity, Obesity + Diab) as the independent variable, while longitudinal comparisons were carried out using *Time* (t0, t6, t12) as the independent variable. DMPs were defined by contrasting coefficients using an empirical Bayes-moderated t-test, such that the set of p-values was adjusted for multiple comparisons using the Benjamini–Hochberg method (FDR < 0.05).

To discover distinct methylation clusters, significant DMPs (0 > 6 and 6 > 12 longitudinal comparisons) were clustered by using Spearman correlation distances to group their scaled methylation values. The optimal number of clusters was determined using the within-cluster sum of squared error method.

### Region-level differential methylation analyses

The “comb-p” method [42] was used to find differentially methylated regions (DMRs) at FDR < 0.05 via the *Enmix* package (v.1.28.2) [37] using default parameters. In order to discover spatially-associated regions of significance, the *limma* p-values from the DMP analyses were fed into the *combp* function. These initial regions were first selected under an FDR threshold, and then final significant DMRs were defined as those with a Sidak-corrected p < 0.05 and containing at least 3 CpG sites. In addition, “mixed” DMRs displaying less than 66% of CpG sites with changes in the same direction were filtered out along with those DMRs whose changes were lower than or equal to 1% of mean methylation value.

### Probe annotation and testing

The *IlluminaHumanMethylationEPICanno.ilm10b4.hg19* package (v.0.6.0) was used to assign each probe to its CGI (CpG Island) and gene location status. Fisher’s exact tests were used to compare statistically differential proportions of annotations and intersections, and odds ratios (ORs) were employed as a measure of the association effect with respect to a particular feature. An appropriate background which included the filtered probes analysed by the EPIC array was used for statistical purposes.

For the annotation of regions, the probes belonging to each region were first individually annotated as described above. A single annotation was then assigned to each region according to the following criteria: (1) for CGI status, “Island” > “N_Shore” > “S_Shore” > “N_Shelf” > “S_Shelf” > “OpenSea”; and (2) for gene locations, “TSS1500” > “TSS200” > “5'UTR” > “1stExon” > “Body” > “ExonBnd” > “3'UTR” > “Intergenic”.

### Pathway enrichment analyses

Pathway enrichment analyses were performed using the *missMethyl* package (v.1.22.0) [43] on the gene sets from the Molecular Signatures Database (MSigDB) [44] accessed via the *msigdbr* package (v.7.2.1). The *gsameth* function was used to interrogate the functionality of the DMPs identified, while the *gsaregion* function was used to analyse DMRs. Both take into account the number of probes mapping to each gene as a bias factor for the enrichment analyses. To visualise pathway enrichment results, several networks of gene-set similarity were built using the *EnrichmentMap* application [45] in *Cytoscape* (v.3.9.1) [46] using the *RCy3* package (v.2.8.1) [47] with the default combined similarity cutoff.

## Results

### Genome-wide profiling of DNA methylation alterations during the first year of life

**Fig. 1:**
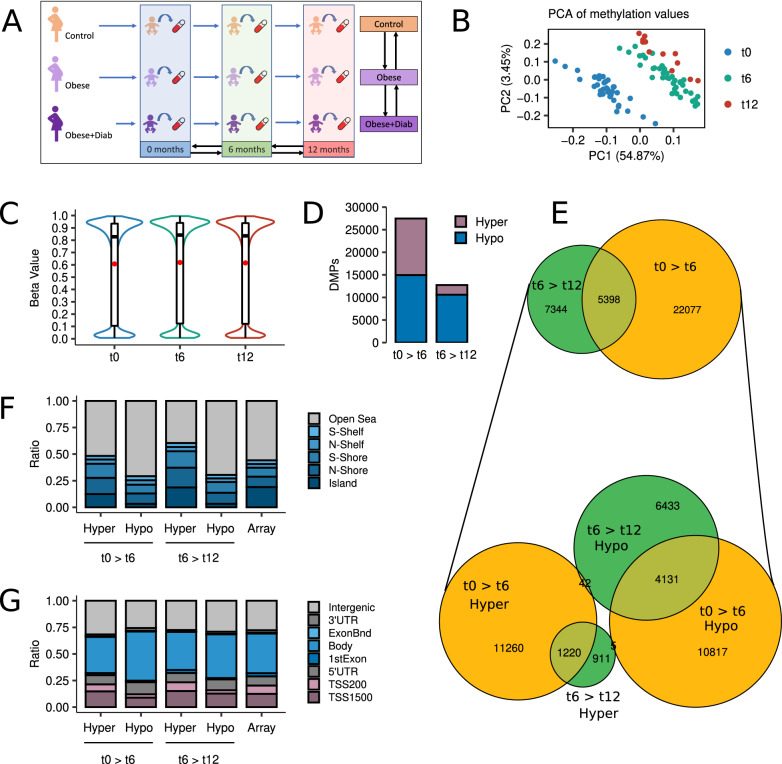
Development is the main origin of methylation changes during the first year of life. **a** Schematic of the study design. **b** Scatter plot showing the PCA of the subjects according to their methylation values at the top 100,000 most variable CpG sites. **c** Violin plots describing the distribution of methylation values at the 772,088 CpGs analysed by time point. **d** Barplots depicting the number of hyper- and hypomethylated DMPs (FDR < 0.05) found in 0 > 6 and 6 > 12 longitudinal comparisons. **e** At the top, a Venn diagram showing the intersections between 0 > 6 and 6 > 12 DMPs. At the bottom, a Venn diagram describing the intersection between hyper- and hypomethylated DMPs at 0 > 6 and 6 > 12 comparisons. **f** Barplots showing the relative distribution of hyper- and hypomethylated DMPs at 0 > 6 and 6 > 12 comparisons according to their CpG island location status. **g** Barplots showing the relative distribution of hyper- and hypomethylated DMPs at 0 > 6 and 6 > 12 comparisons according to their gene location status. Rightmost bars represent the background distribution considering all 772,088 probes analysed

To characterize DNA methylation alterations that occur during the first year of development, we studied the methylome of whole blood samples from a paediatric cohort of 39 subjects consisting of children born to mothers suffering from obesity or obesity with GDM during pregnancy, and healthy controls (Table 1; Fig. 1A; Additional file 2: Table S1). We performed longitudinal measurements throughout the first year of their infants’ lives (0, cord blood; 6 and 12 months, peripheral blood; total N = 90) using MethylationEPIC arrays to profile 772,088 CpG sites in differential methylation analyses (see “Methods”), in which we considered cell-type composition biases as we found variation in proportions, especially significant during the first six months of life (Additional file 1: Fig. S1).

**Table 1:** Summary of clinical information

Group	Control (C)			Obese (Ob)			Obese + Diabetes (ObDia)		
Time point	t0	t6	t12	t0	t6	t12	t0	t6	t12
Number of subjects	13	13	4	15	15	4	11	11	4
Sex (M/F)	8/5	8/5	2/2	11/4	11/4	3/1	6/5	6/5	2/2
Maternal pregnancy BMI, mean (SD)	22.58 (1.42)	22.58 (1.42)	22.62 (1.28)	35.46 (5.25)	35.46 (5.25)	37.57 (7.61)	32.87 (2.33)	32.87 (2.33)	32.92 (0.66)
Child weight/g, mean (SD)	3202 (404)	7585 (1032)	9078 (248)	3435 (508)	7867 (1098)	10,465 (675)	3404 (409)	8106 (1368)	10,640 (1087)
Child height/cm, mean (SD)	49.23 (2.07)	65.31 (3.22)	72.25 (2.87)	49.17 (1.90)	65.47 (1.95)	75.00 (1.29)	49.14 (1.60)	66.09 (5.25)	75.88 (2.66)

Based on maternal BMI/GDM, subjects are classified into controls (C) (maternal BMI < 30), obese (Ob) (maternal BMI > 30) and obese (maternal BMI > 30) with gestational diabetes (ObDia). Children were followed-up at birth (t0), six months (t6) and one year of life (t12)

First, we sought to describe global aspects of DNA methylation alterations through an exploratory principal component analysis (PCA) (Fig. 1B) and were able to confirm that developmental processes are those with the deepest impact on the paediatric methylome. Nonetheless, we did not observe noticeable differences in global methylation values during the first year of child development (Fig. 1C), so we focused on identifying differentially methylated CpG sites (DMPs). To this end, we employed empirical Bayes moderated t tests across the different age groups (FDR < 0.05; see “Methods”) and discovered abundant DNA methylation changes during development from birth to 6 months (27,475 DMPs) and, to a lesser extent, from 6 to 12 months of age (12,742 DMPs) (Fig. 1D; Additional file 3: Table S2). The direction of DNA methylation changes was well balanced from birth to 6 months in that 14,953 DMPs were found to be hypomethylated (~ 54%) while 12,522 were hypermethylated (~ 46%). Conversely, this balance disappeared in the following 6 months, and at 12 months 10,606 DMPs (~ 83%) were found to be hypomethylated whereas only 2136 DMPs were hypermethylated (~ 17%).

Next, we proceeded to analyse the degree of overlap between those CpG sites that were differentially methylated between 0 and 6 months and between 6 and 12 months (Fig. 1E), finding that a large proportion of DMPs at 0 > 6 months were also altered at 6 > 12 months (Fisher’s test P < 0.001, OR = 25). Moreover, those common changes massively preserved their direction of change at both longitudinal comparisons (hyper: Fisher’s test P < 0.001, OR = 89; hypo: Fisher’s test P < 0.001, OR = 44) (Fig. 1E), although they were noticeably stronger in the 0 > 6 period (Additional file 1: Fig. S2).

When we studied the genomic distribution of the DMPs identified (Fig. 1F, G), we determined that hypermethylated CpGs tended to be located at CpG island-associated loci (0 > 6 Fisher’s test P < 0.001, OR = 1.18; 6 > 12 Fisher’s test P < 0.001, OR = 1.93) and promoters (TSS 0 > 6 Fisher’s test P < 0.01, OR = 1.07; TSS 6 > 12 Fisher’s test P < 0.001, OR = 1.20). In fact, this result is particularly important since the array background is already enriched in CpG sites that are mainly located at island and promoter regions. Inversely, hypomethylated CpGs tended to be enriched at open sea regions (0 > 6 Fisher’s test P < 0.001, OR = 1.94; 6 > 12 Fisher’s test P < 0.001, OR = 1.81), gene bodies (0 > 6 Fisher’s test P < 0.001, OR = 1.46; 6 > 12 Fisher’s test P < 0.001, OR = 1.18) and 5’UTR regions (0 > 6 Fisher’s test P < 0.001, OR = 1.37; 6 > 12 Fisher’s test P < 0.001, OR = 1.20), revealing that the direction of the methylation change is intrinsically dependent on the genomic context of its target.

### Functional dynamics of DNA methylation alterations during the first year of life

**Fig. 2:**
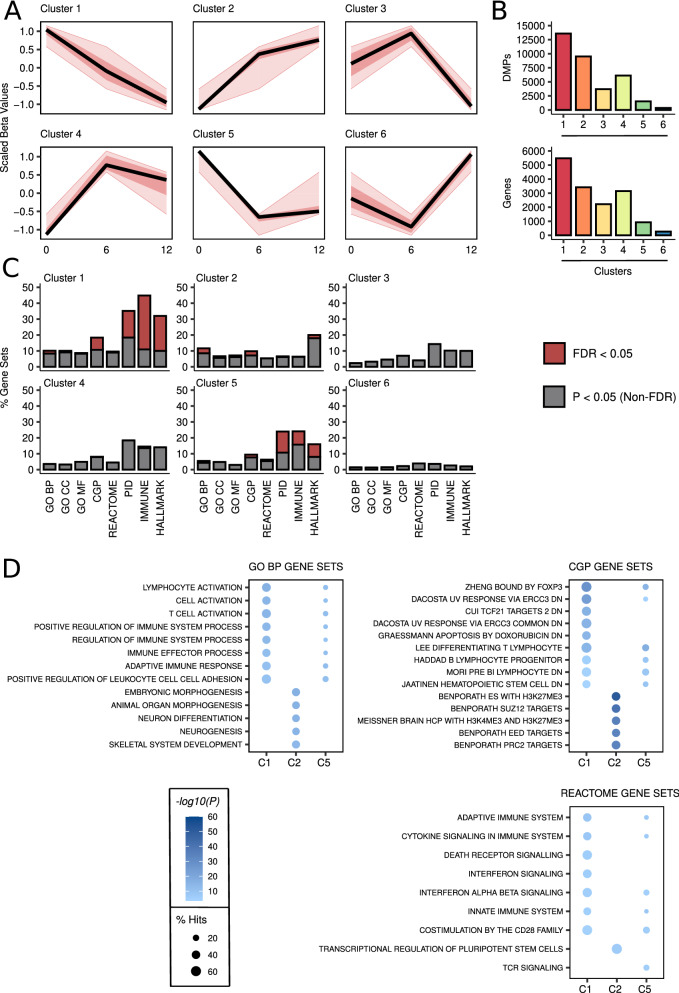
Hyper- and hypomethylation changes affect different functional targets during development. **a** Line plots depicting the scaled methylation patterns at 0, 6 and 12 months of the six clusters identified. **b** At the top, barplots showing the number of DMPs for each cluster. At the bottom, barplots of the number of associated genes by cluster. **c** Barplots showing the proportion of significant gene sets (P < 0.05, grey; FDR < 0.05, red) found in the enrichment analyses for each cluster and type of MSigDb database. **d** Bubble plots showing the top 5 most significant GO BP, CGP and REACTOME gene sets (FDR < 0.05) for clusters 1, 2 and 5

In order to study the functional context in which DNA methylation changes accumulate during development, we clustered the aforementioned DMPs on the basis of their methylation patterns at 0, 6 and 12 months (see “Methods”). We found six distinct clusters (Fig. 2A) which differed in size and numbers of annotated genes, the number of DMPs and associated genes being correlated (Fig. 2B). The two main clusters (1 and 2) reflected gradual longitudinal hypo- and hypermethylation alterations over the first year of life, while clusters 4 and 5 underwent, respectively, marked hyper- and hypomethylation changes from 0 to 6 months. Finally, clusters 3 and 6 grouped directionally opposite changes for both longitudinal comparisons.

Next, we performed pathway enrichment analyses against several databases from the MSigDB collection to ascertain which of these clusters were functionally relevant. We concluded that clusters 1, 2 and 5 were the most informative according to the numbers of statistically significant gene sets found (P < 0.05; FDR < 0.05) (Fig. 2C). The main roles of the genes associated with the hypermethylated CpGs from cluster 2 were related to developmental processes, being targets of repression by Polycomb (*PRC2*, *SUZ12*) and part of the pluripotency regulation mechanisms that control cell differentiation (Fig. 2D). We then explored the genes related to these pathways that showed a very high number of associated DMPs (Additional File 1: Figure S3), and found several examples with relevant developmental roles, such as transcription factors *NFIX* and *TBX1*, growth factors *WNT10A* and *NRG2*, hormone *CALCA* and cadherin *CDH23*, amongst others.

In contrast, hypomethylated CpG-linked genes from clusters 1 and 5 were mainly involved in immune system activation and maturation pathways (Fig. 2D). In fact, we found a high level of overlap between the significant gene sets belonging to clusters 1 and 5, but nothing between these and cluster 2 (Additional file 1: Fig. S4; CGP C1–C5 Fisher’s test P < 0.001, OR = 42; GO BP C1–C5 Fisher’s test P < 0.001, OR = 296; REACTOME C1–C5 Fisher’s test P < 0.001, OR = 195). When we analysed the genes that showed a high number of associated hypo-DMPs, we distinguished many targets that regulate hematopoietic differentiation, especially the development, selection and maturation of T and NK cells (Additional File 1: Figure S5), including transcription factors (*FOXP1*, *ETS1*, *HIVEP2* and *HIVEP3*), cell adhesion proteins (*CYTH1*, *ITGAL*, *ITGB2*, HLA-E), apoptotic genes (*BCL-2*, TNF family) and epigenetic modifiers (PRDM histone methyltransferases). Therefore, during early postnatal development, the functionality of the affected genes is clearly defined by the direction of the DNA methylation changes.

### Maternal obesity and gestational diabetes during pregnancy alter the methylomic landscape of the offspring

Once we had described the DNA methylation alterations during the first year of development, we sought to answer several questions: (1) To what extent could maternal influence during pregnancy affect the methylome of the offspring? (2) Could those putative methylome changes be preserved at least during the first year of life? To address these questions, we defined three groups in our paediatric cohort: (a) children born to mothers with obesity during pregnancy (Ob); (b) children born to mothers with obesity and GDM during pregnancy (ObDia); (c) children born to healthy mothers (C).

**Fig. 3:**
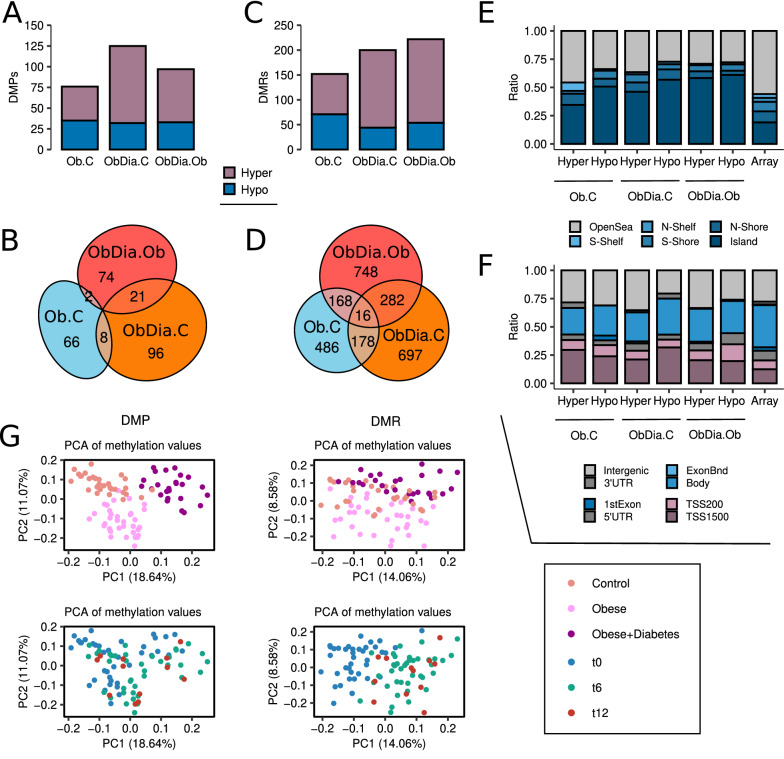
Obesity-mediated maternal influence during pregnancy alters the methylome of offspring during the first year of life. **a** Barplots depicting the number of hyper- and hypomethylated DMPs (FDR < 0.05) found in the Ob.C, ObDia.C and ObDia.Ob cross-sectional comparisons. **b** Venn diagram showing the intersection between hyper- and hypomethylated DMPs found in the Ob.C, ObDia.C and ObDia.Ob cross-sectional comparisons. **c** Barplots depicting the number of hyper- and hypomethylated DMRs (FDR < 0.05) found in the Ob.C, ObDia.C and ObDia.Ob cross-sectional comparisons. **d** Venn Diagram showing the intersection between hyper- and hypomethylated CpGs from DMRs found in the Ob.C, ObDia.C and ObDia.Ob cross-sectional comparisons. **e** Barplots depicting the relative distribution of hyper- and hypomethylated DMRs from the Ob.C, ObDia.C and ObDia.Ob comparisons according to their CpG island locations status. **F** Barplots showing the relative distribution of hyper- and hypomethylated DMRs from the Ob.C, ObDia.C and ObDia.Ob comparisons according to their gene location status. Rightmost bars represent the background distribution considering all 772,088 probes analysed. **g** On the left, scatter plot showing the PCA of the subjects according to their methylation values at Ob.C, ObDia.C, ObDia.Ob DMPs. On the right, scatter plot representing the PCA of the subjects according to their methylation values at CpGs from Ob.C, ObDia.C, ObDia.Ob DMRs

First, we again performed differential methylation analyses to uncover DMPs (FDR < 0.05) that were able to distinguish our experimental groups throughout the first year of life. We detected moderate DNA methylation changes across all comparisons (Ob.C, ObDia.C, ObDia.Ob) (Fig. 3A; Additional file 4: Table S3). When we studied the degree of overlap between the three biological comparisons (Fig. 3B), we observed large enrichments in shared DMPs (Ob.C U ObDia.C Fisher’s test P < 0.001, OR = 782; ObDia.C U ObDia.Ob Fisher’s test P < 0.001, OR = 2130; Ob.C U ObDia.Ob Fisher’s test P < 0.001, OR = 220), indicating that these metabolic processes target common loci. Overlaps between the comparisons were also observed when separating hyper- and hypo-DMPs (Additional file 1: Fig. S6A).

Next, to increase our power to detect DNA methylation alterations with functional implications for the molecular physiopathology of obesity, we performed differential methylation analyses at the regional level to discover differentially methylated regions (DMRs; FDR < 0.05; see “Methods”). With this method, we detected even more numerous alterations across all comparisons (Fig. 3C; Additional file 5: Table S4). To analyse the regional overlap between the three biological comparisons (Fig. 3D), we studied the shared CpGs belonging to the DMRs, finding large enrichments for all of them (Ob.C U ObDia.C Fisher’s test P < 0.001, OR = 234; ObDia.C U ObDia.Ob Fisher’s test P < 0.001, OR = 284; Ob.C U ObDia.Ob Fisher’s test P < 0.001, OR = 207), thus indicating a shared epigenetic footprint associated with these metabolic processes. Interestingly, when we separated the regions into hyper- or hypo-DMRs, we observed that the intersection between ObDia.Ob and Ob.C DMRs disappeared, suggesting that GDM also produces directionally-specific DNA methylation alterations which may not be associated with obesity (Additional file 1: Fig. S6B).

By exploring the genomic distribution of the DMRs, we concluded that both hyper- and hypo-DMRs were clearly located at CpG islands (Fig. 3E) (Ob.C hyper Fisher’s test P = 0.073, OR = 1.51; Ob.C hypo Fisher’s test P < 0.001, OR = 2.49; ObDia.C hyper Fisher’s test P < 0.001, OR = 2.20; ObDia.C hypo Fisher’s test P < 0.001, OR = 3.38; ObDia.Ob hyper Fisher’s test P < 0.001, OR = 3.08; ObDia.Ob hypo Fisher’s test P < 0.001, OR = 3.30) and promoter regions (Fig. 3F) (Ob.C hyper Fisher’s test P < 0.001, OR = 2.44; Ob.C hypo Fisher’s test P < 0.01, OR = 2.01; ObDia.C hyper Fisher’s test P < 0.01, OR = 1.60; ObDia.C hypo Fisher’s test P < 0.01, OR = 2.48; ObDia.Ob hyper Fisher’s test P = 0.067, OR = 1.40; ObDia.Ob hypo Fisher’s test P < 0.001, OR = 3.40). As anticipated, the regional-level analysis of DNA methylation alterations allowed us to discover candidates for obesity-mediated maternal influence, which were concentrated at regulatory sequences and thus could have potential functional implications.

We also confirmed, via PCA, how these DNA methylation biomarkers were able to distinguish our phenotypes of at the DMP level (Fig. 3G, top left) and, more subtly, at the DMR level (Fig. 3G, top right). Interestingly, we observed that these biomarkers were also affected by developmental-associated changes during the first year of life, as was revealed by grouping the subjects by time point, whereby the main component (PC1) tended to separate t0 subjects from t6 and t12 subjects within each maternal group in the PCA with DMPs (Fig. 3G, bottom left), and across all groups when using DMRs (Fig. 3G, bottom right). Thus, both DMPs and DMRs captured developmental epigenetic alterations, especially during the first six months of life, thereby establishing a relationship between maternal metabolic condition and early childhood development.

### Maternal DNA methylation biomarkers are enriched in functional signatures related to metabolic pathways

**Fig. 4:**
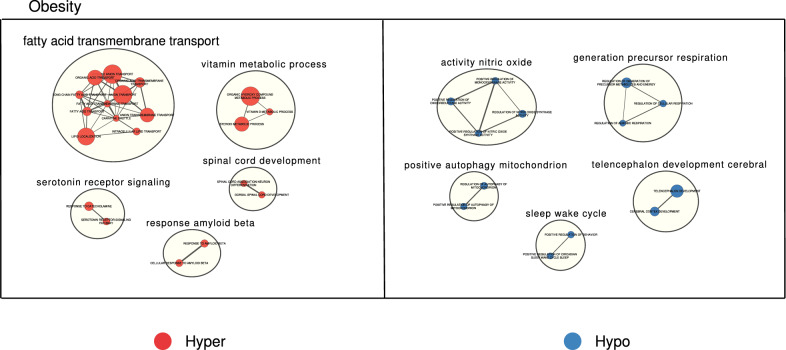
Maternal obesity generates DNA methylation signatures in offspring that are related to the metabolism of organic compounds, fatty acids and mitochondria. Network showing the similarity between the pathways found enriched (unadjusted P < 0.05) in the significant DMR analyses involving the Ob.C comparison. The clusters are coloured to show hyper- (red) and hypomethylation (blue) alterations

After establishing the existence of maintained DNA methylation alterations in offspring according to the maternal condition, we sought to explore their functional features and whether they could constitute epigenetic signatures of metabolic interest. Consequently, we performed pathway enrichment analyses against the Gene Ontology Biological Process database to clarify whether the Ob.C hyper- and hypo- DMRs were concentrated in genes relevant for the molecular physiopathology of obesity. Using the top 25 most-significant obesity-associated ontologies (unadjusted P < 0.05), we built a network through gene-set similarity in which we observed clusters of highly similar pathways of great interest (Fig. 4), in spite of the limited number of DMRs involved in the analysis. Hypermethylated DMRs were strikingly concentrated in genes involved in the transport of organic compounds, fundamentally fatty acids, as well as in the metabolism of vitamins and steroids (Fig. 4, left panel). On the other hand, hypomethylated DMRs were located at genes involved in mitochondrial metabolism through aerobic respiration, mitochondrial autophagy and nitric oxide production (Fig. 4, right panel). In addition, DMR alterations within the obesity with gestational diabetes group (ObDia.C) were also enriched (unadjusted P < 0.05) in developmental processes for hypermethylation and metabolic pathways for hypomethylation (Additional file 1: Fig. S7).

Next, we explored several gene candidates affected by the regional DNA methylation alterations linked to obesity and GDM some of which could also present development-associated alterations. (Additional file 1: Fig. S8). Some transporter genes of organic compounds (*ATP11A*, *CPT1B*, *SLC38A4*) were consistently hypermethylated in the Ob and ObDia groups at all time points and, furthermore, underwent hypermethylation in development between 0 and 6–12 months. In contrast, the *TTYH1* transporter gene was hypermethylated in the Ob and ObDia groups, but did not exhibit hypermethylation during development. We also found diabetes-specific biomarkers as *SLC35F3* gene, which was exclusively hypermethylated in the ObDia group. Regarding hypomethylated DMRs (Additional file 1: Fig. S9), they involved genes linked to metabolic control of diabetes such as *FN3K*, *RPH3AL* and *HOX, HDAC4*. These candidates were mainly hypomethylated in the Ob and ObDia groups at all time points and did not undergo appreciable changes during development.

### The interplay between developmental and maternal-derived DNA methylation alterations in offspring

**Fig. 5:**
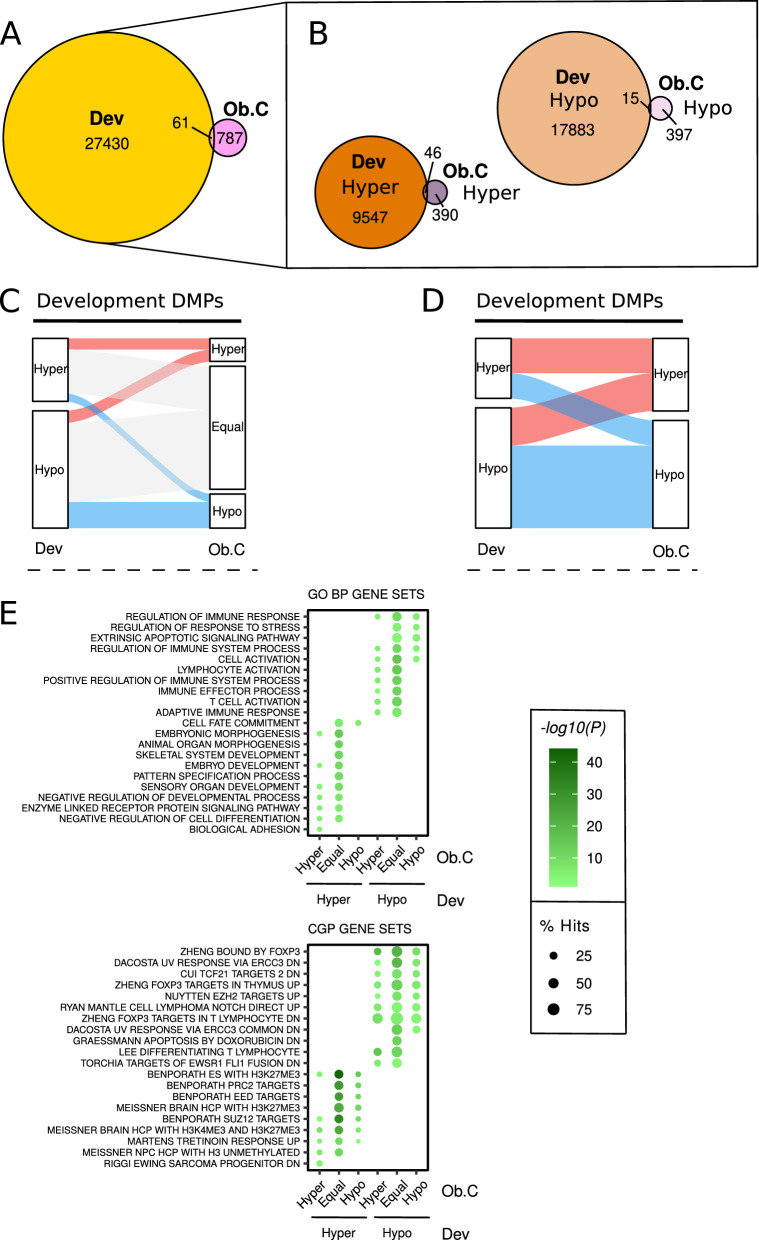
Maternal obesity globally affects postnatal development pathways. **a** Venn diagram showing the intersection between the DMPs that change at least in one longitudinal comparison preserving the direction of the change (Dev) and the CpGs found in the Ob.C DMRs. **b** Venn diagrams depicting the overlaps between aforementioned CpGs, separated by the direction of the methylation change. **c** Sankey diagram describing the distribution of Dev DMPs when considering the changes between Ob and C subjects. **d** Sankey diagram showing the distribution of those Dev DMPs that experience hyper- and hypo- changes between Ob and C subjects. **e** Bubble plots showing the top 5 most significant GO BP and CGP gene sets (FDR < 0.05) for the Dev DMPs based on their methylation patterns between Ob and C subjects

After defining the genomic and functional features of the infants’ DNA methylation alterations, we integrated the specific DNA methylation alterations coming from developmental epigenetic remodelling with those produced by maternal metabolic condition during pregnancy to gain insight into the interaction between both processes. We first studied whether development-associated CpGs (defined as those differentially methylated at least in one longitudinal comparison (0 > 6 or 6 > 12) preserving the direction of methylation change during the first year of life; Dev CpGs: 27,491 CpGs; 9593 hyper; 17,898 hypo) were particularly affected by maternal metabolic alterations, and, interestingly, we observed a significant enrichment between Dev CpGs and those from maternal obesity-associated DMRs (Fig. 5A, Fisher’s test P < 0.001, OR = 2.10), particularly in the case of the hypermethylation changes (hyper: Fisher’s test P < 0.001, OR = 9.42; hypo: Fisher’s test P = 0.097, OR = 1.59) (Fig. 5B). We next classified Dev CpGs into three groups based on the mean methylation change that Ob children experienced compared to controls: Hyper (> 1% gain), Hypo (> 1% loss), Equal (≤ 1% change). The majority of Dev CpGs did not change between Ob and C children (18,774 CpGs, ~ 68%), but 5209 (~ 19%) suffered hypomethylation while 3508 CpGs underwent hypermethylation (~ 13%) (Fig. 5C). When we excluded those developmental changes that remain Equal for Ob.C comparison, we observed an extremely significant association between the direction of the methylation changes in development and obesity (Fisher’s test P < 0.001, OR = 3.02) (Fig. 5D). In sum, these results support the hypothesis that maternal obesity during pregnancy is able to alter postnatal development in a longitudinal fashion.

In the case of gestational diabetes, we did not find evidence for enrichment between ObDia.C alterations and Dev CpGs in general (Fisher’s test P = 0.237, OR = 0.81) (Additional File 1: Fig. S10A), though we again found that hypermethylation processes showed significant enrichment of common changes (hyper: Fisher’s test P < 0.01, OR = 2.04; hypo: Fisher’s test P = 0.335; OR = 1.33) (Additional file 1: Fig. S10B). By grouping Dev CpGs in categories based on average DNA methylation change of ObDia.C comparison (± 1% change), we observed 18,503 Equal CpGs (~ 67%), 6014 Hyper (~ 22%) and 2974 Hypo (~ 11%) (Additional file 1: Fig. S10C). Unlike previous analyses for Ob.C, the proportion of hypermethylation changes compared to hypomethylation alterations increased with the appearance of GDM. Considering only those developmental CpGs that change with ObDia condition, we found a strong association between the direction of the methylation changes in development and obesity with GDM (Fisher’s test P < 0.001, OR = 5.55; Additional file 1: Fig. S10D). Therefore, our evidence suggests that maternal metabolic condition during pregnancy influences early child development though DNA methylation alterations which usually intensify the molecular changes that occur during normal development.

Next, we characterized the molecular pathways enriched in DNA methylation alterations during postnatal development, taking into account the effect of obesity and obesity with GDM. Enrichment analyses for both the GO BP and CGP MSigDB databases (FDR < 0.05) revealed that maternal-influenced Dev CpGs are located in the same pathways than those that did not suffer appreciable changes by maternal condition (Fig. 5E, Additional file 1: Fig. S10E). This result reinforces the idea that the maternal effect on early development tends to be global and untargeted, for both hypermethylation and hypomethylation processes.

**Fig. 6:**
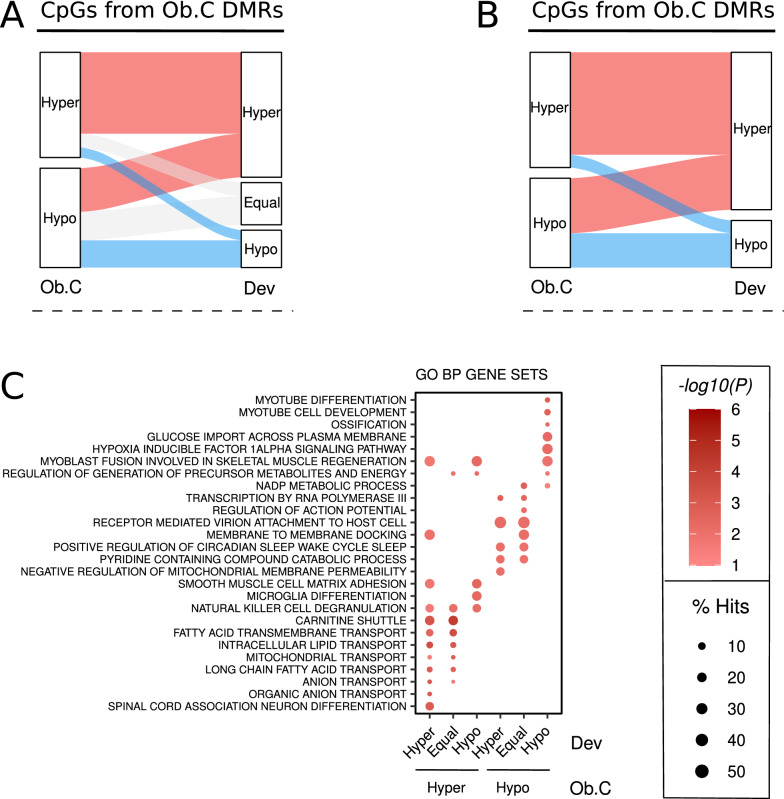
Development modifies the methylation of obesity biomarkers in offspring without altering their functionality. **a** Sankey diagram describing the distribution of CpGs coming from Ob.C DMRs when considering their methylation differences during development. **b** Sankey diagram showing the distribution of Ob.C DMR CpGs excluding those that do not change during development. **c** Bubble plots showing the top 5 most significant GO BP and CGP gene sets (unadjusted P < 0.05) for the Ob.C DMR CpGs based on their methylation patterns across development

Finally, we also investigated if maternal-condition CpGs from Ob.C and ObDia.C DMRs were particularly targeted by developmental changes found. To this end, we classified those CpGs in three groups based on the average DNA methylation change that occurred between t12 and t0: Hyper (> 1% gain), Hypo (> 1% loss), Equal (≤ 1% change). In the case of Ob.C DMR CpGs, the majority suffered from hypermethylation changes during development (518 CpGs, ~ 61%), while 155 CpGs experienced hypomethylation (~ 18%) and 175 remained without noticeable changes (~ 21%) (Fig. 6A). We observed the same pattern for ObDia.C DMR CpGs (Hyper: 633, ~ 54%; Hypo: 214, ~ 18%; Equal: 326, ~ 28%) (Additional file 1: Fig. S11A). Again, the association between the direction of methylation based on the maternal condition and the postnatal development was very strong for Ob.C DMR CpGs (Fisher’s test P < 0.001, OR = 5.00; Fig. 6B) and ObDia.C DMR CpGs (Fisher’s test P < 0.001, OR = 1.97; Additional file 1: Fig. S11B). When we evaluated the GO BP pathways that were enriched in DNA methylation alterations (unadjusted P < 0.05), we determined that DMR CpGs that suffered developmental changes did not show any clear differential pattern with those that were not altered, for both Ob.C (Fig. 6C) and ObDia.C (Additional file 1: Fig. S11C) comparisons. Therefore, although DMR CpGs captured developmental alterations, the molecular functions associated to maternal effect on offspring prevailed over any consequence related to the direction of the developmental DNA methylation change.

## Discussion

In this work, we have analysed the paediatric methylome across the first year of life in order to establish the existence of epigenetic signatures that reflect the maternal metabolic condition during pregnancy on the offspring beyond birth. To this end, we employed a cohort formed by longitudinal whole blood samples at 0, 6 and 12 months, whose methylome was profiled using Illumina Infinium MethylationEPIC BeadChip arrays. With this strategy, we first characterized those DNA methylation alterations produced during postnatal development, defining the first six months of life as the most dynamic for epigenetic remodelling. In addition, we observed that a significant proportion of the altered loci continued to experience changes until the age of 12 months, preserving the direction of the effect, which highlights their importance in achieving a correct postnatal development. Likewise, we performed cross-sectional studies to infer systemic DNA methylation alterations that allow to distinguish children born to mothers suffering from obesity or obesity with GDM during pregnancy from infants born to healthy controls. Our results revealed that there are DNA methylation biomarkers at both single CpG positions and genomic regions that track these maternal differences in pregnancy at least during the first year of infants’ lives. These DNA methylation alterations based on the maternal condition were concentrated at genes that recapitulated metabolic pathways of transport of fatty acids, mitochondrial bioenergetics and several developmental processes. Although an important part of these biomarkers experienced parallel alterations across the first year of life, the functionality of the involved genes remained in the same metabolic signatures, highlighting the consistency of these biomarkers during development. Interestingly, we found that maternal influence during pregnancy was able to alter postnatal development, especially those changes that implied gain of methylation. In addition, more subtle influences in development confirmed that maternal obesity tended to intensify development processes at the methylation level, in a global way, without apparently targeting any specific developmental process. Therefore, the results presented in this manuscript provide a systematic, comprehensive analysis of the epigenetic dynamics associated with the early postnatal development, the prenatal intrauterine conditioning and the interactions between both processes in a longitudinal fashion.

Regarding postnatal development, our results are in line with previous studies that establish the first 5 years of life [22] as the most critical for epigenetic remodelling, especially during the first 3 years [48]. The uncovering of the epigenetic relevance of the first six months of life could lead to new studies that point the importance of earlier lifestyle interventions on adult health, especially at the cardiovascular level. From a functional viewpoint, the genes that are involved in maturation processes of the hematopoietic compartment undergo a regulation programme mediated by hypomethylation changes, thus being a potential source of transcriptional activation, as has been previously reported [49]. On the other hand, loci involved in gene regulatory networks of embryonic development concentrate hypermethylation signatures in their promoter regions, consolidating Polycomb-mediated gene repression programmes once developmental processes have concluded [50, 51]. These results agree with previous longitudinal studies carried out from birth up to 10 years [22, 48]. All in all, early postnatal development is tightly regulated at the epigenetic level according to the direction of the methylation changes.

Using our longitudinal design, we performed a comprehensive epigenetic profiling at the single-CpG level to uncover reliable DNA methylation biomarkers of maternal obesity (with or without GDM) in infant blood samples. Importantly, these alterations were maintained beyond birth across the first year of life. Moreover, detecting DNA methylation changes at the regional level constituted a better option for discovering alterations enriched at regulatory sequences and thus with potential functional implications. Strikingly, our results revealed a clear enrichment of the alterations in functions important for the molecular physiopathology of obesity. For instance, hypermethylated regions affected important deregulated genes in cardiometabolic diseases, the majority related to the transport of organic molecules and fatty acids. In obese subjects, *CPT1B* promoter hypermethylation has been associated with diminished muscular expression in response to lipids leading to a reduced ability to oxidize fatty acids [52]. The *SLC38A4* amino acid transporter, which is crucial for the placental nutrition of the embryo, is overexpressed in the placentas from human foetuses with macrosomia [53], while its knockout causes foetal weight reduction in mice [54]. Several polymorphisms in this transporter gene are also linked to the appearance of hyperglycaemia [55] even in the placenta of normal-weight newborns [56]. Similarly, Soranzo and colleagues have shown that *ATP11A* is significantly associated with the levels of HbA1c, which is used to monitor diabetes [57]. The *SLC35F3* gene, a diabetes-specific biomarker, is a thiamine transporter that exhibits polymorphisms related to increased blood pressure and potential higher risk of hypertension [58, 59]. When we considered the hypomethylated regions, we found genes such as *FN3K*, which has been associated with HbA1clevels [57]. In addition, the *RPH3AL* and *HOX* genes are known to suffer DNA methylation alterations during adipogenesis in cells from obese patients with and without type II diabetes [60]. We also observed alterations in epigenetic modifiers such as *HDAC4*, whose mutation impairs β-cell function and insulin secretion, leading to a non-autoimmune paediatric diabetes [61].

In this work, we also seek to draw attention to the interplay between intrauterine conditioning and postnatal development. Importantly, obesity-mediated epigenetic alterations are maintained regardless of the developmental changes that can occur concurrently at those CpG sites. In fact, those DNA methylation biomarkers that are also modified during development are still related to the same metabolic functional pathways, providing robust evidence for the conservation of these epigenetic signatures with time. From a developmental viewpoint, we observed that maternal obesity with or without GDM tends to magnify the DNA methylation changes which occur with time, although opposite dynamics are also observed. Further longitudinal studies will be needed to ascertain if maternal metabolic condition causes an acceleration of developmental processes, at least from an epigenetic perspective. All in all, these results provide support for the notion that the maternal metabolic influence during pregnancy can affect the epigenetic features of early development, with hitherto unexplored consequences for future health. Ideally, public health interventions should focus on controlling the maternal metabolic status during pregnancy, but at the same time, our results point that the first six months of infant’s lives are crucial for an adequate postnatal development.

That said, our observations present limitations. First, individual genetic traits could explain why our obese-mediated DNA methylation changes are longitudinally maintained during the first year of life [62]. In addition, although our results support the existence of an obesity-mediated intrauterine effect on the childhood methylome, we cannot rule out that the maintenance of the DNA methylation changes across the first year of life is the result of early-life lifestyle factors. Nevertheless, our study clearly positions epigenetic mechanisms as the molecular link that explains the environmental maternal influence in the offspring. Other works that use methylation arrays also support this conclusion in a non-longitudinal fashion, showing that gestational diabetes is associated with methylation changes of metabolic genes in placenta [25, 26, 63, 64] and blood samples from newborns, children and adolescents [65–70]. Fewer studies have addressed associations between DNA methylation alterations and maternal obesity, those that exist mainly using only cord blood samples from newborns [71–74]. Finally, despite the fact that our study does not allow us to infer direct functional consequences, detecting these changes in blood indicates that maternal influence causes a systemic effect in the offspring through epigenetic footprints that are preserved beyond birth.

## Conclusions

Altogether, in this work we have described DNA methylation alterations in whole blood cells of offspring born to mothers suffering from obesity (with or without GDM) during pregnancy, on the basis of a longitudinal follow-up. Importantly, this foetal reprogramming is maintained during the first year of life and displays epigenetic signatures enriched in metabolic pathways, thus suggesting a link between the maternal intrauterine environment and the offspring’s genome function. Moreover, some of these alterations are observed to interact with the epigenetic reprogramming occurring during early-life development. The definition of DNA methylation biomarkers in the offspring which are associated with maternal condition may be of value for estimating the risk for the development of cardiometabolic diseases, but further studies will be needed to assess their feasibility. From a paediatric perspective, we foresee that our results could pave the way to the design of earlier interventions that limit the appearance of cardiometabolic events in adult life. Thus, our longitudinal design could be a baseline for future studies aimed at exploring the potential functional implications of these epigenetic footprints on intergenerational health.

## Supplementary Information

**Additional file 1:** Supplementary Information and Supplementary Figures S1–11. **Figure S1. **Barplots depicting the average blood cell-type composition for each combination of group (Control, Obese, Obese+Diab) and time point (t0, t6, t12), inferred via the Houseman algorithm from methylation data. **Figure S2**. Violin plot showing the magnitude of change of those DMPs common to common 0>6 and 6>12 which had the same direction of change at both time points. The magnitude of change is measured as the absolute β-value difference in the average methylation values of each CpG between the longitudinal groups. The P-value from a Wilcoxon rank sum test is shown. **Figure S3**. Boxplots showing examples of genes that accumulate a high number of hypermethylation alterations (FDR<0.05) during the first year of development. **Figure S4**. Upset plots depicting intersections of significant results (FDR<0.05) for Chemical and Genetic Perturbations (CGP), Gene Ontology Biological Process (GOBP) and Reactome (REACT) gene set enrichment analyses. **Figure S5**. Boxplots showing examples of genes that accumulate a high number of hypomethylation alterations (FDR<0.05) during the first year of development. **Figure S6**. Venn diagrams indicating (A) the overlap of DMPs between all comparisons (Ob.C, ObDia.C, ObDia.Ob) in terms of hypermethylation DMPs and hypomethylation (FDR<0.05) and (B) the overlap of CpGs belonging to DMRs (Sidak P<0.05) between the same comparisons. **Figure S7**. Network showing the similarities found in the ObDia.C comparison with respect to the pathways found enriched (unadjusted P<0.05) in the significant DMR analyses. Blue clusters relate to hypomethylation alterations and red clusters to hypermethylated DMRs. **Figure S8**. Boxplots showing examples of DMRs that are hypermethylated in the obesity and/or obesity+diabetes groups (Sidak P-value<0.05). **Figure S9**. Boxplots showing some examples of DMRs that are hypomethylated in the obesity and/or obesity+diabetes groups (Sidak P-value<0.05). **Figure S10**. (a) Venn diagram showing the intersection between the DMPs that change at least in one longitudinal comparison preserving the direction of the change (Dev) and the CpGs found in the ObDia.C DMRs. (b) Venn diagrams depicting the overlaps between aforementioned CpGs, separated by the direction of the methylation change. (c) Sankey diagram describing the distribution of Dev DMPs when considering the changes between ObDia and C subjects. (d) Sankey diagram showing the distribution of those Dev DMPs that experience hyper- and hypo- changes between ObDia and C subjects. (e) Bubble plots showing the top 5 most significant GO BP and CGP gene sets (FDR<0.05) for the Dev DMPs based on their methylation patterns between ObDia and C subjects. **Figure S11.** (a) Sankey diagram describing the distribution of CpGs coming from ObDia.C DMRs when considering their methylation differences during development. (b) Sankey diagram showing the distribution of ObDia.C DMR CpGs excluding those that do not change during development. (c) Bubble plots showing the top 5 most significant GO BP and CGP gene sets (unadjusted P<0.05) for the ObDia.C DMR CpGs based on their methylation patterns across development.

**Additional file 2: Table S1.** Expanded clinical information related to the subjects.

**Additional file 3: Table S2.** List and details of the DMPs (FDR<0.05) found in the longitudinal analyses (0>6; 6>12).

**Additional file 4: Table S3.** List and details of the DMPs (FDR<0.05) found in the cross-sectional analyses (Ob.C; ObDia.C; ObDia.Ob).

**Additional file 5: Table S4.** Lists and details of the DMRs (Sidak-corrected P<0.05) found in the cross-sectional analyses (Ob.C; ObDia.C; ObDia.Ob).

## Data Availability

All data generated during this study are included in the published article and its supplementary files. The raw IDAT and processed data are also available in the ArrayExpress public repository under accession E-MTAB-12728.

## Abbreviations

BMI: Body mass index
GDM: Gestational diabetes mellitus
DNA: Deoxyribonucleic acid
CpG: Cytosine nucleotide followed by a guanine nucleotide
GCT: Glucose challenge test
OGTT: Glucose tolerance test
WHO: World Health Organisation
IDAT: Intensity data
SNP: Single nucleotide polymorphism
MAF: Minor allele frequency
SBE: Single base extension
DMPs: Differentially methylated probes
t0/t6/t12: Timepoint (0/6/12 months of life)
FDR: False discovery rate
DMRs: Differentially methylated regions
CGI: CpG Island
OR: Odds ratio
MSigDB: Molecular Signature DataBase
P: P-value
PCA: Principal component analysis
*PRC2*: Polycomb repressive complex 2
*SUZ12*: SUZ12 polycomb repressive complex 2 subunit
*NFIX*: Nuclear factor IX
*TBX1*: T-Box transcription factor 1
*WNT10A*: Wingless-type MMTV integration site family, member 10A
*NRG2*: Neuregulin 2
*CALCA*: Calcitonin related polypeptide alpha
*CDH23*: Cadherin related 23
CGP: Chemical and genetic perturbations (gene sets)
GO BP: Gene ontology biological process (gene sets)
*FOXP1*: Forkhead box P1
*ETS1*: ETS proto-oncogene 1, transcription factor
*HIVEP2*: Human immunodeficiency virus type I enhancer-binding protein 2
*HIVEP3*: Human immunodeficiency virus type I enhancer-binding protein 3
*CYTH1*: Cytohesin 1
*ITGAL*: Integrin subunit alpha L
*ITGB2*: Integrin subunit beta 2
*HLA-E*: Major histocompatibility complex, class I, E
*BCL-2*: BCL2 apoptosis regulator
TNF: Tumor necrosis factor
PRDM: PR/SET domain
C: Control group
Ob: Obese group
ObDia: Obese + Diabetes group
Ob.C: Obese group vs Control group (comparison)
ObDia.C: Obese + Diabetes group vs Control group (comparison)
ObDia.Ob: Obese + Diabetes group vs Obese group (comparison)
*ATP11A*: ATPase Phospholipid Transporting 11A
*CPT1B*: Carnitine palmitoyltransferase 1B
*SLC38A4*: Solute carrier family 38 member 4
*TTYH1*: Tweety family member 1
*SLC35F3*: Solute carrier family 35 member F3
*FN3K*: Fructosamine 3 kinase
*RPH3AL*: Rabphilin 3A like (without C2 domains)
*HOX*: Homeobox
*HDAC4*: Histone deacetylase 4
HbA1c: Glycated hemoglobin
Dev: Development-associated CpGs
