# Supplementary material for: Maternal obesity and gestational diabetes reprogram the methylome of offspring beyond birth by inducing epigenetic signatures in metabolic and developmental pathways
Source: Cardiovasc Diabetol. 2023 Mar 4;22:44. doi: 10.1186/s12933-023-01774-y (PMC9985842; doi:10.1186/s12933-023-01774-y)
Supplement: Supplementary file 1 — Additional file 1: Supplementary Information and Supplementary Figures S1–11. Figure S1. Barplots depicting the average blood cell-type composition for each combination of group (Control, Obese, Obese+Diab) and time point (t0, t6, t12), inferred via the Houseman algorithm from methylation data. Figure S2. Violin plot showing the magnitude of change of those DMPs common to common 0>6 and 6>12 which had the same direction of change at both time points. The magnitude of change is measured as the absolute β-value difference in the average methylation values of each CpG between the longitudinal groups. The P-value from a Wilcoxon rank sum test is shown. Figure S3. Boxplots showing examples of genes that accumulate a high number of hypermethylation alterations (FDR<0.05) during the first year of development. Figure S4. Upset plots depicting intersections of significant results (FDR<0.05) for Chemical and Genetic Perturbations (CGP), Gene Ontology Biological Process (GOBP) and Reactome (REACT) gene set enrichment analyses. Figure S5. Boxplots showing examples of genes that accumulate a high number of hypomethylation alterations (FDR<0.05) during the first year of development. Figure S6. Venn diagrams indicating (A) the overlap of DMPs between all comparisons (Ob.C, ObDia.C, ObDia.Ob) in terms of hypermethylation DMPs and hypomethylation (FDR<0.05) and (B) the overlap of CpGs belonging to DMRs (Sidak P<0.05) between the same comparisons. Figure S7. Network showing the similarities found in the ObDia.C comparison with respect to the pathways found enriched (unadjusted P<0.05) in the significant DMR analyses. Blue clusters relate to hypomethylation alterations and red clusters to hypermethylated DMRs. Figure S8. Boxplots showing examples of DMRs that are hypermethylated in the obesity and/or obesity+diabetes groups (Sidak P-value<0.05). Figure S9. Boxplots showing some examples of DMRs that are hypomethylated in the obesity and/or obesity+diabetes groups (Sidak P-value<0.05). [file 12933_2023_1774_MOESM1_ESM.docx]

**Additional file**

- Additional file Tables 1-4
- Additional file Figures 1-11

The raw IDAT and processed data are also available in the ArrayExpress public repository under accession E-MTAB-12728**.**

**Additional file table legends**

**Table S1.** Expanded clinical information related to the subjects.

**Table S2.** List and details of the DMPs (FDR<0.05) found in the longitudinal analyses (0>6; 6>12).

**Table S3.** List and details of the DMPs (FDR<0.05) found in the cross-sectional analyses (Ob.C; ObDia.C; ObDia.Ob).

**Table S4.** Lists and details of the DMRs (Sidak-corrected P<0.05) found in the cross-sectional analyses (Ob.C; ObDia.C; ObDia.Ob).

**Additional file figures**


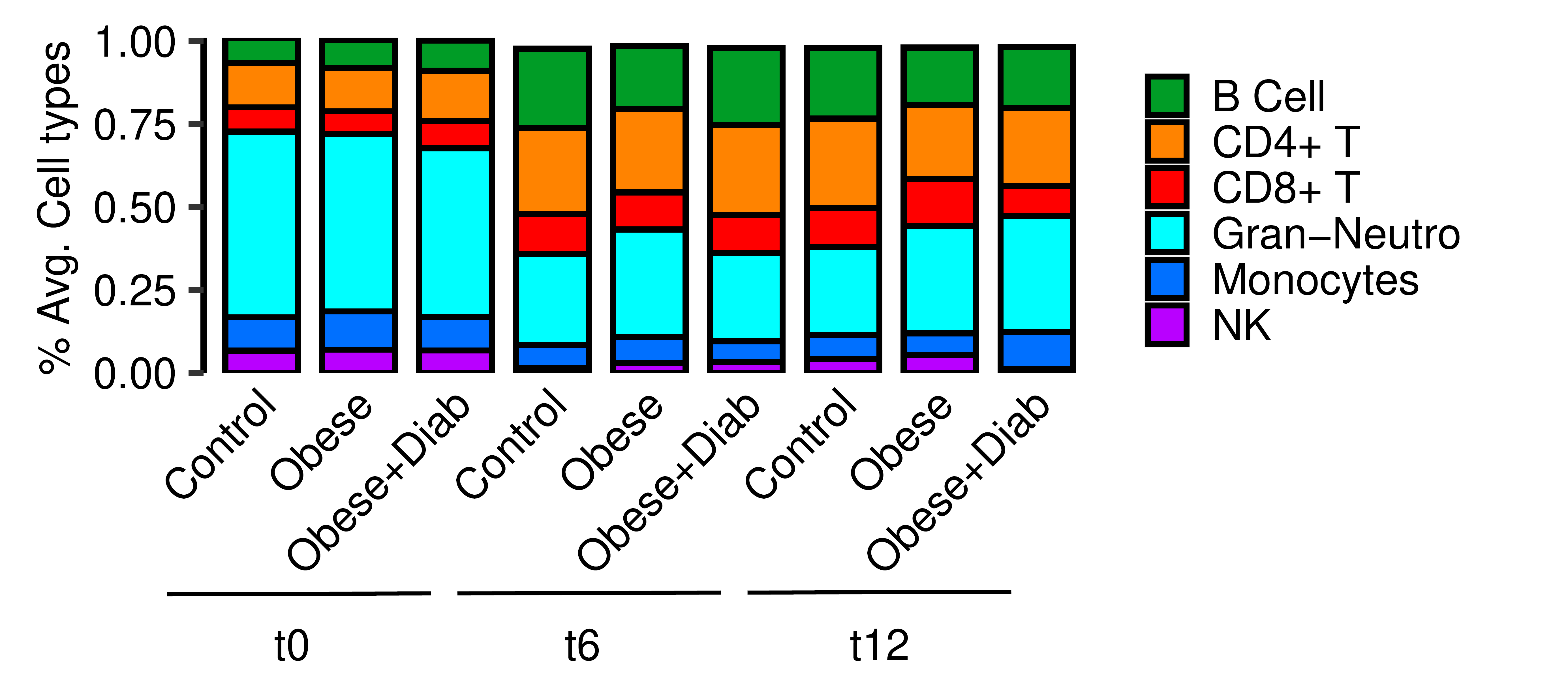


**Figure S1.** Barplots depicting the average blood cell-type composition for each combination of group (Control, Obese, Obese+Diab) and time point (t0, t6, t12), inferred via the Houseman algorithm from methylation data.

**
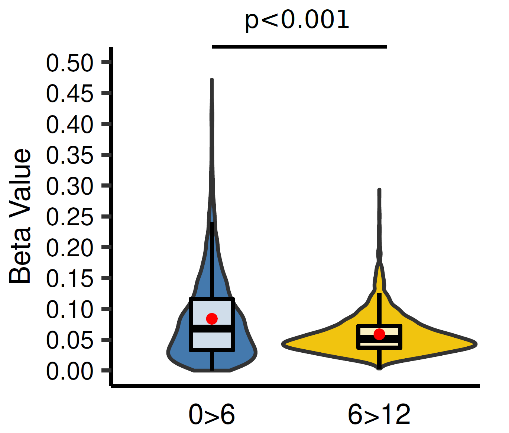
**

**Figure S2**. Violin plot showing the magnitude of change of those DMPs common to common 0>6 and 6>12 which had the same direction of change at both time points. The magnitude of change is measured as the absolute β-value difference in the average methylation values of each CpG between the longitudinal groups. The P-value from a Wilcoxon rank sum test is shown.


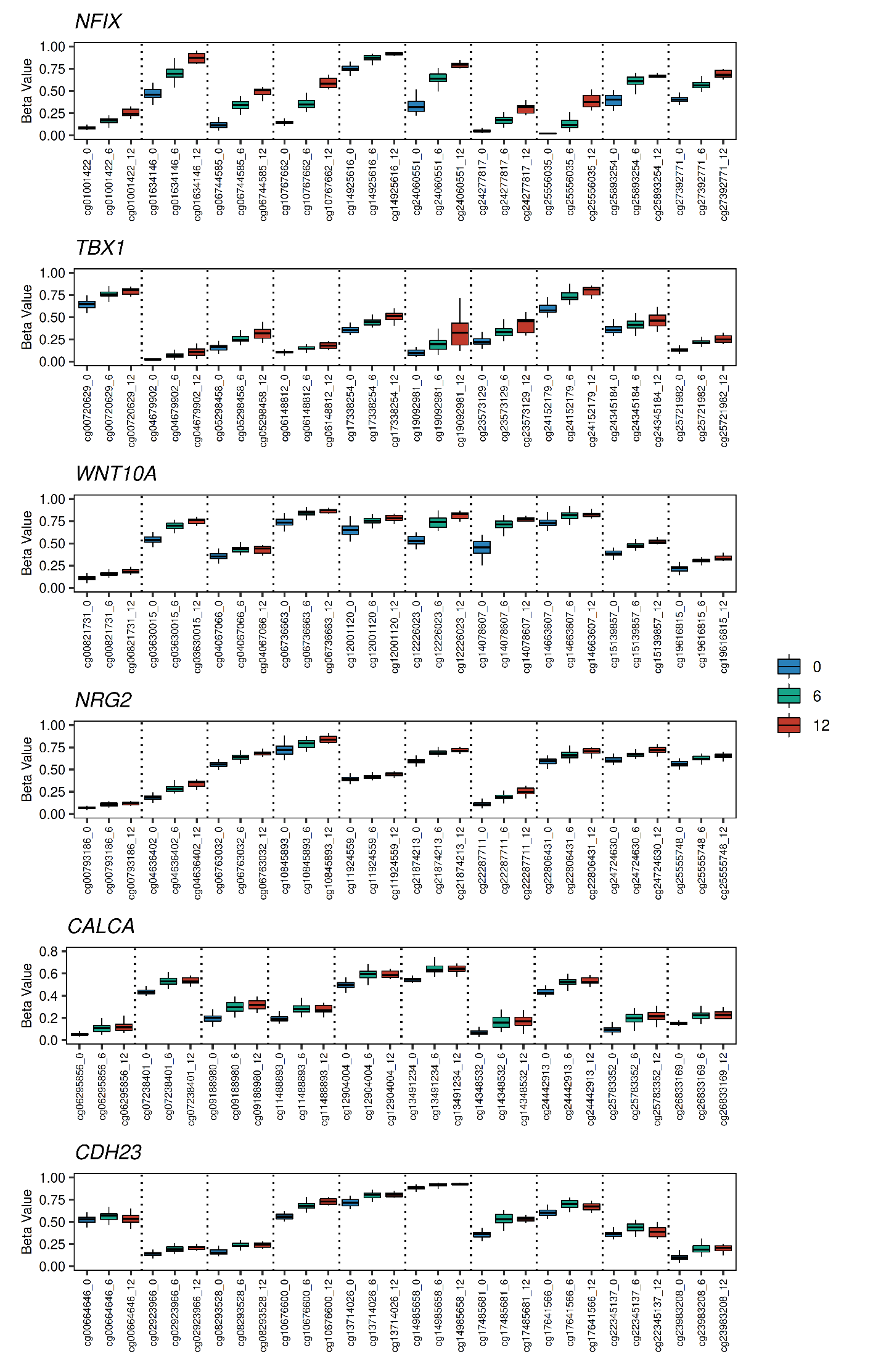


**Figure S3**. Boxplots showing examples of genes that accumulate a high number of hypermethylation alterations (FDR<0.05) during the first year of development.


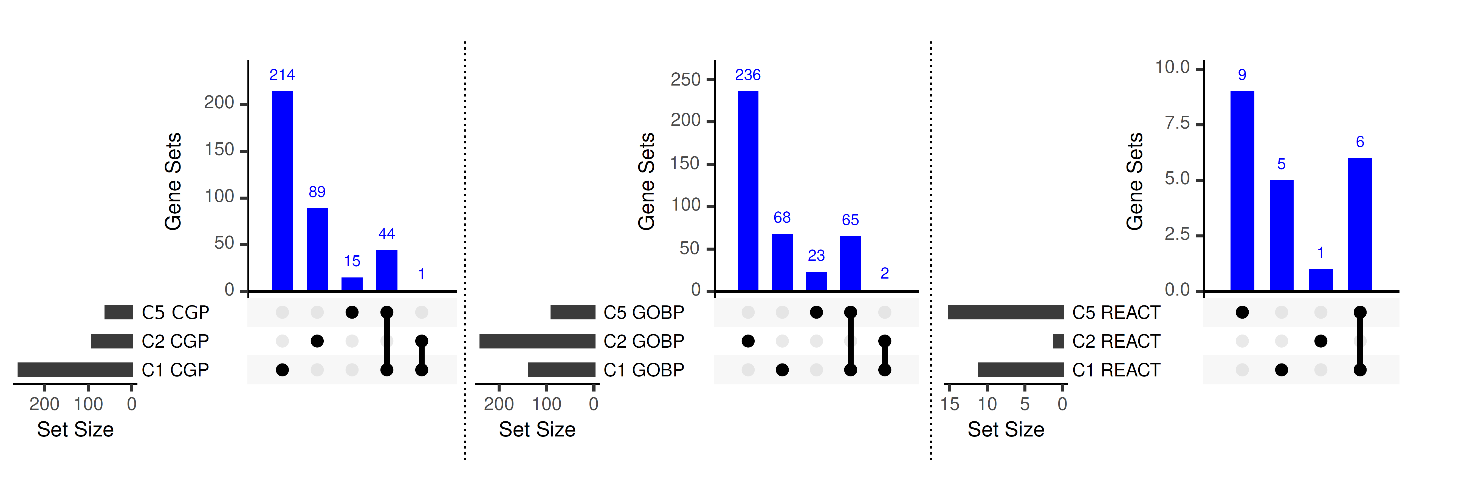


**Figure S4**. Upset plots depicting intersections of significant results (FDR<0.05) for Chemical and Genetic Perturbations (CGP), Gene Ontology Biological Process (GOBP) and Reactome (REACT) gene set enrichment analyses.


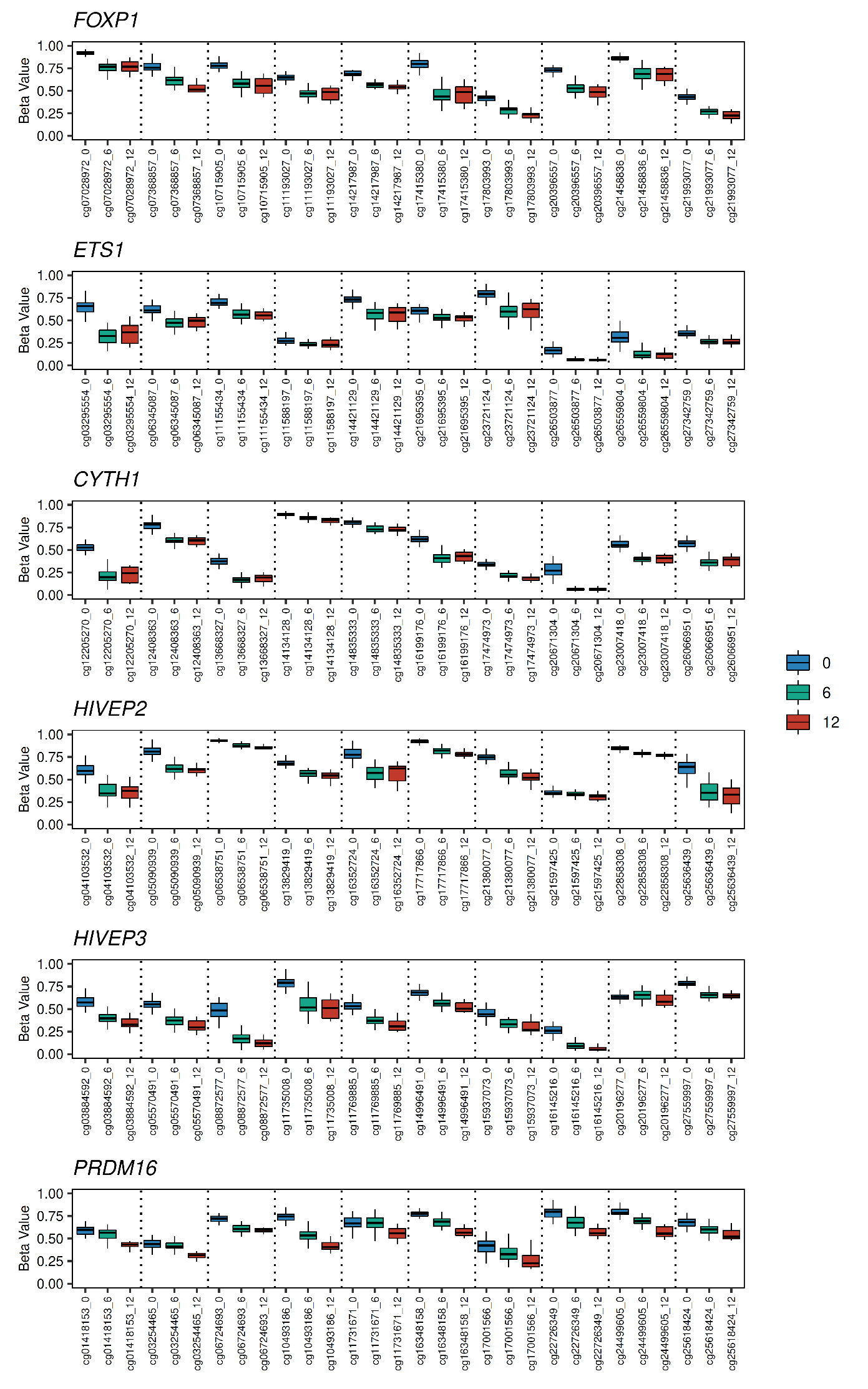


**Figure S5**. Boxplots showing examples of genes that accumulate a high number of hypomethylation alterations (FDR<0.05) during the first year of development.


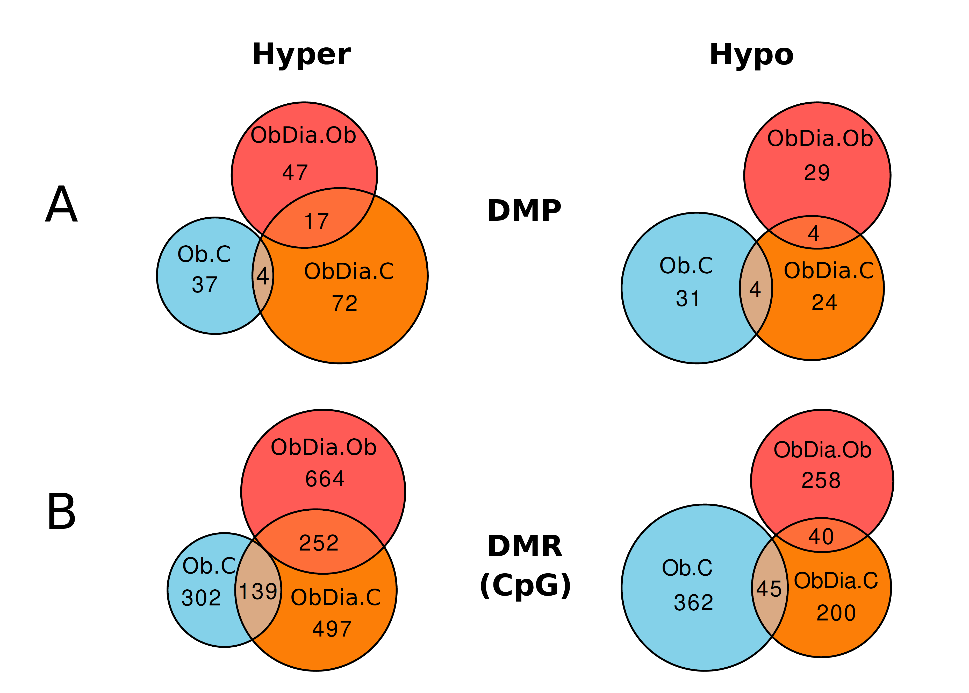


**Figure S6**. Venn diagrams indicating (A) the overlap of DMPs between all comparisons (Ob.C, ObDia.C, ObDia.Ob) in terms of hypermethylation DMPs and hypomethylation (FDR<0.05) and (B) the overlap of CpGs belonging to DMRs (Sidak P<0.05) between the same comparisons.


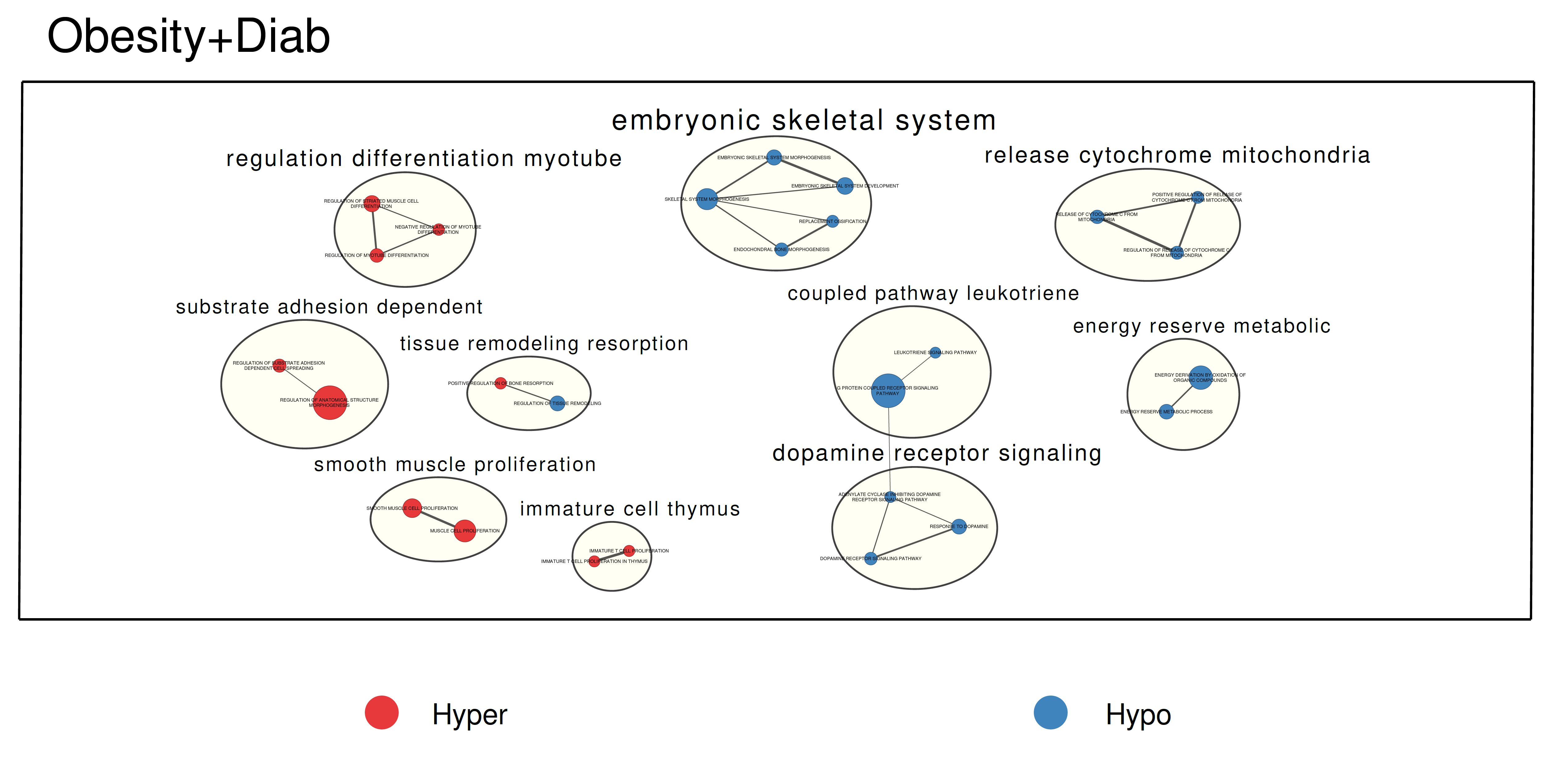


**Figure S7**. Network showing the similarities found in the ObDia.C comparison with respect to the pathways found enriched (unadjusted P<0.05) in the significant DMR analyses. Blue clusters relate to hypomethylation alterations and red clusters to hypermethylated DMRs.


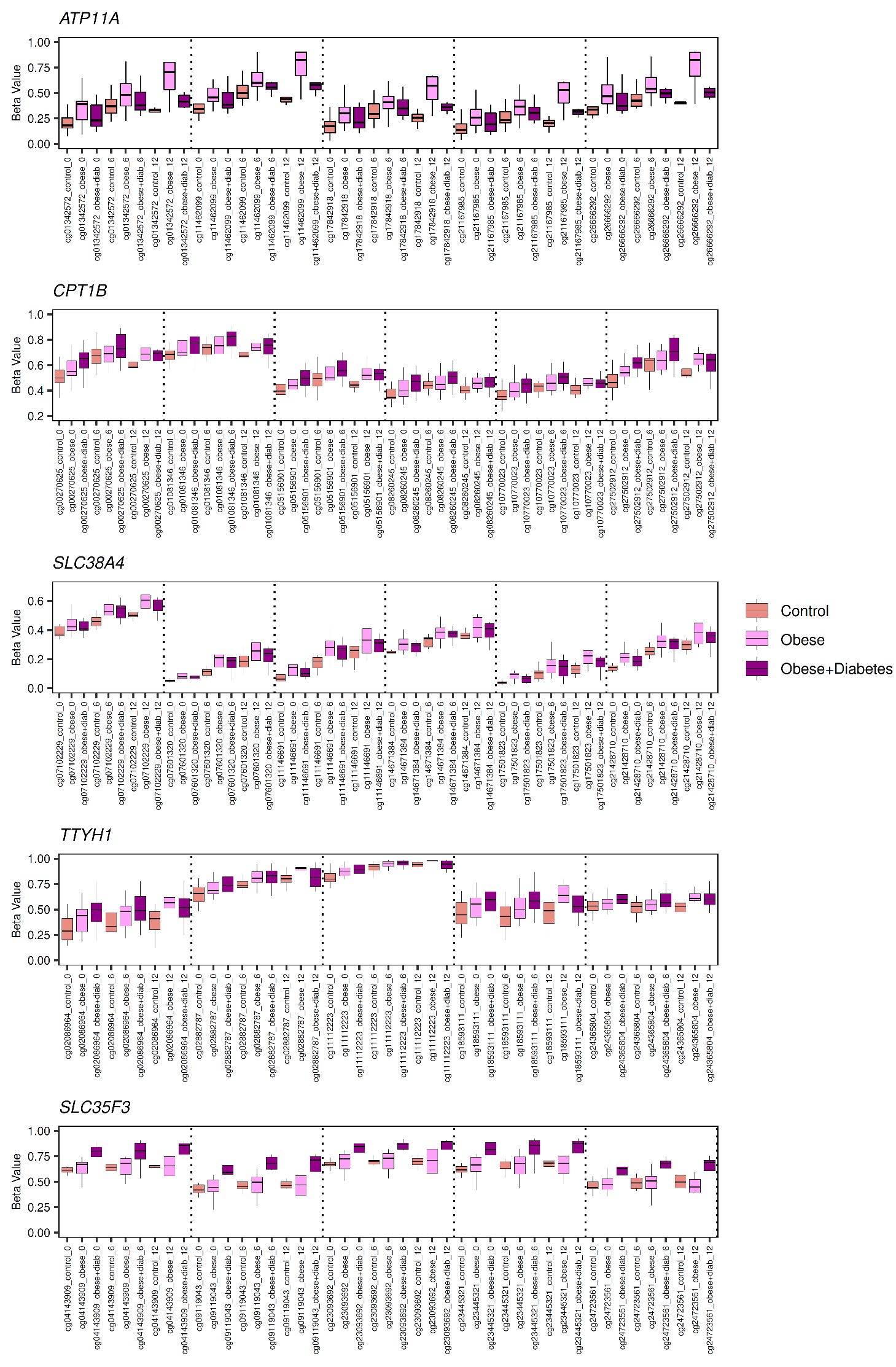


**Figure S8**. Boxplots showing examples of DMRs that are hypermethylated in the obesity and/or obesity+diabetes groups (Sidak P-value<0.05).


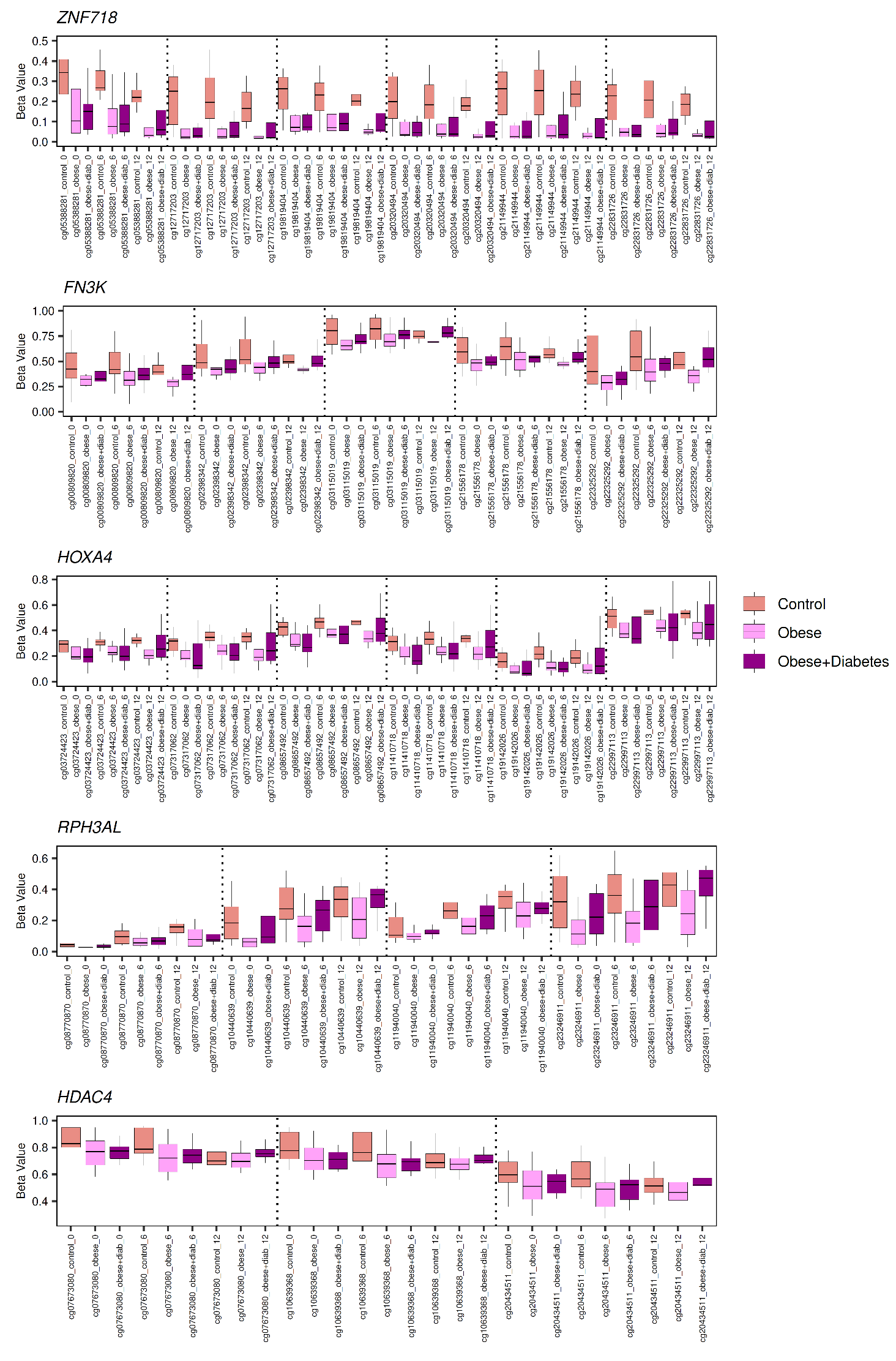


**Figure S9**. Boxplots showing some examples of DMRs that are hypomethylated in the obesity and/or obesity+diabetes groups (Sidak P-value<0.05).


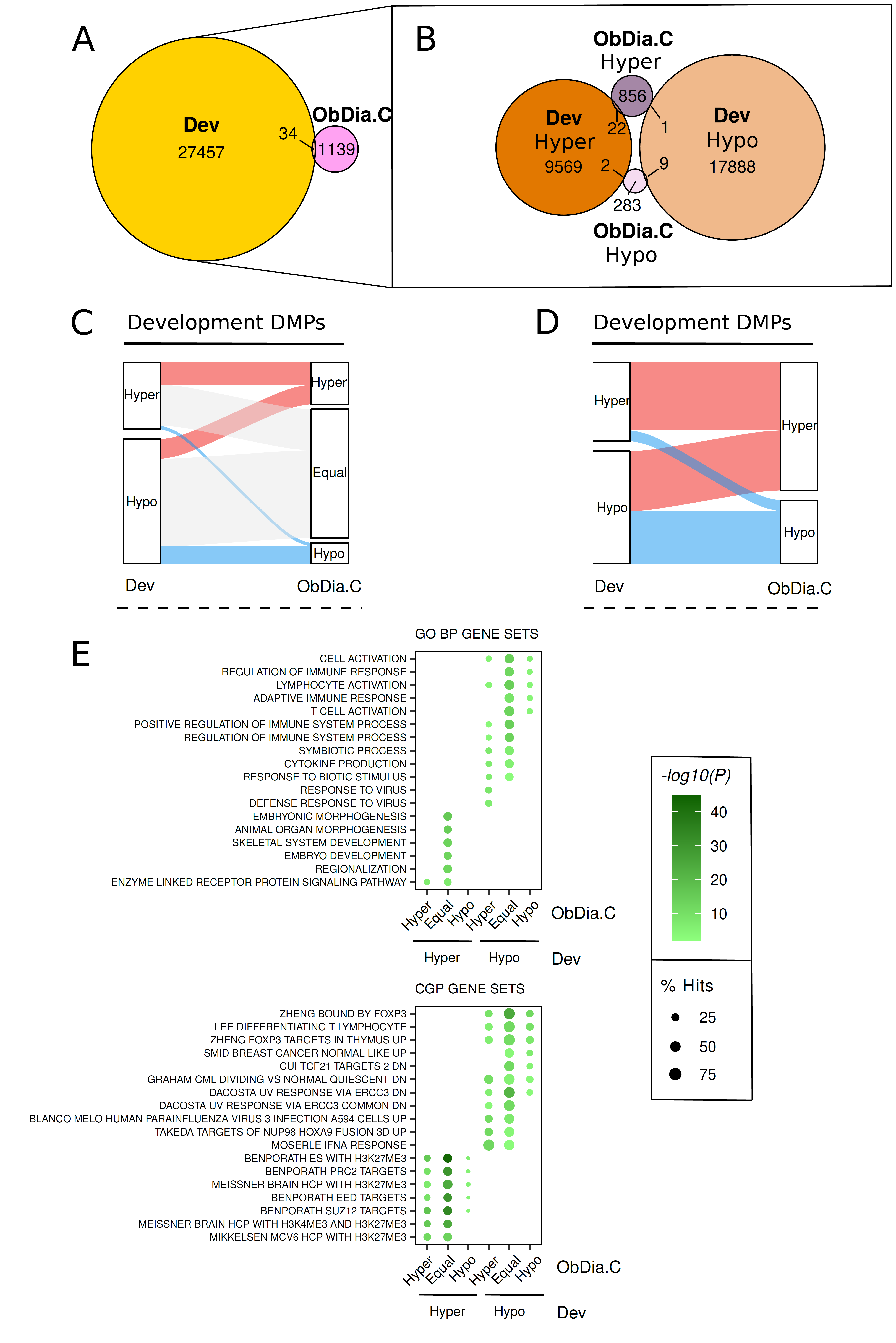


**Figure S10**. (a) Venn diagram showing the intersection between the DMPs that change at least in one longitudinal comparison preserving the direction of the change (Dev) and the CpGs found in the ObDia.C DMRs. (b) Venn diagrams depicting the overlaps between aforementioned CpGs, separated by the direction of the methylation change. (c) Sankey diagram describing the distribution of Dev DMPs when considering the changes between ObDia and C subjects. (d) Sankey diagram showing the distribution of those Dev DMPs that experience hyper- and hypo- changes between ObDia and C subjects. (e) Bubble plots showing the top 5 most significant GO BP and CGP gene sets (FDR<0.05) for the Dev DMPs based on their methylation patterns between ObDia and C subjects.


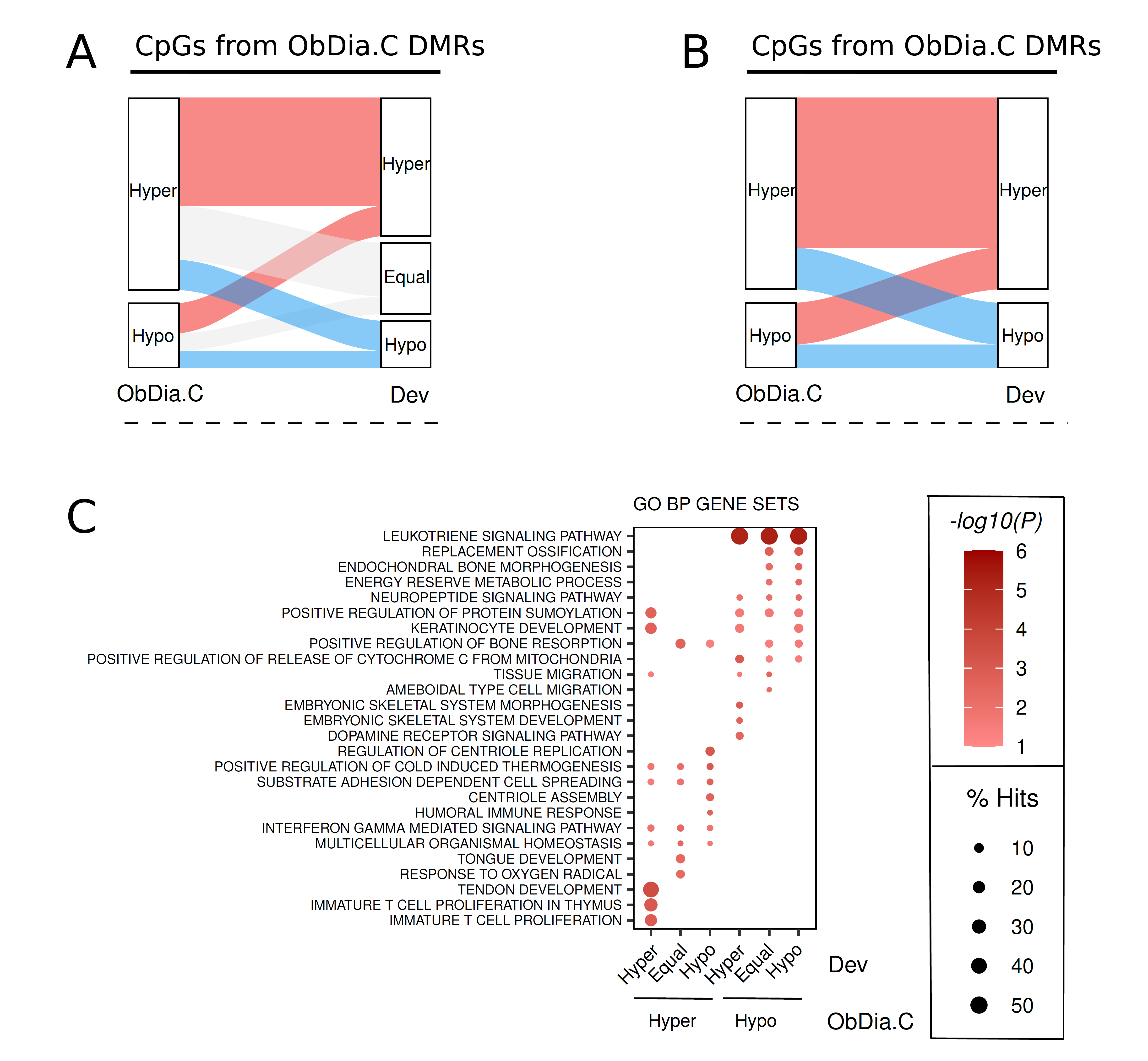


**Figure S11.** (a) Sankey diagram describing the distribution of CpGs coming from ObDia.C DMRs when considering their methylation differences during development. (b) Sankey diagram showing the distribution of ObDia.C DMR CpGs excluding those that do not change during development. (c) Bubble plots showing the top 5 most significant GO BP and CGP gene sets (unadjusted P<0.05) 971 for the ObDia.C DMR CpGs based on their methylation patterns across development.
